# Exploring *Leishmania infantum* cathepsin as a new molecular marker for phylogenetic relationships and visceral leishmaniasis diagnosis

**DOI:** 10.1186/s12879-019-4463-8

**Published:** 2019-10-28

**Authors:** Ryan Emiliano da Silva, Bruna Matarucco Sampaio, Renata Tonhosolo, Andrea Perei ra da Costa, Luiz Eduardo da Silva Costa, Fernanda Ap. Nieri-Bastos, Márcia Aparecida Sperança, Arlei Marcili

**Affiliations:** 1 Departamento de Medicina Veterinária Preventiva e Saúde Animal, Faculdade de Medicina Veterinária e Zootecnia, Universidade de São Paulo, Av. Prof. Dr. Orlando Marques de Paiva, 87, São Paulo, SP 05508-270 Brazil; 2 Faculdade de Medicina, Universidade Santo Amaro, São Paulo, SP Brazil; 3 Ciência Animal, Universidade Estadual do Maranhão, São Luís, MA Brazil; 4 Centro de Ciências Naturais e Humanas, Universidade Federal do ABC, São Bernardo do Campo, SP Brazil; 5 Medicina Veterinária e Bem estar animal, Universidade Santo Amaro, São Paulo, SP Brazil

**Keywords:** *Leishmania (Leishmania) infantum*, Cysteine proteases, Cathepsin L-*like*, Molecular diagnosis

## Abstract

**Background:**

*Leishmania infantum*, the etiological agent of visceral leishmaniasis, is a neglected zoonosis that requires validation and standardization of satisfactory diagnostic methodologies. Thus, the aim of the present study was to evaluate the effectiveness of cathepsin L-*like* protease as a target for making molecular diagnoses and as a phylogenetic marker enabling to understand the intraspecies variations and evolutionary history of *L. infantum* in Brazil.

**Methods:**

We used 44 isolates of *L. infantum*. The cathepsin L-*like* gene fragments were amplified, sequenced, manually aligned and analyzed using inference methods. The sequences generated were used to search and design oligonucleotide primers to be used in reactions specific to the target parasite.

**Results:**

The cathepsin L-*like* gene did not show any intraspecies variability among the isolates analyzed. The pair of primers proposed amplified the target deoxyribonucleic acid (DNA) of *L. infantum* isolates and were effective for DNA amplification at concentrations of as low as 10^− 11^ ng/μl. The proposed marker did not present cross-reactions with other hemoparasites. When used for making the diagnosis in a panel of clinical samples from dogs, a positivity rate of 49.03% (102/208) was obtained, versus 14.42% (30/208) for a ribosomal internal transcribed spacer (ITS) marker. In samples from sandflies, the rate was 6.25% and from humans, 14.28%.

**Conclusion*s*:**

The results described in this work allow us to infer that CatLeish-PCR is a sensitive and specific marker for use in diagnostic trials of *L. infantum* and in clinical and epidemiological surveys.

## Background

*Leishmania (Leishmania) infantum* is a flagellate protozoon with a heterogenic cycle that belongs to the genus *Leishmania*, which is in the family Trypanosomatidae of the order Kinetoplastida [1]. This parasite is the etiological agent for visceral leishmaniasis, a zoonosis with worldwide distribution. Most of the cases are concentrated in Bangladesh, Nepal, India, South Sudan and Brazil. The development of this parasite comprises two morphological forms, whose main differences are the position of the kinetoplast in relation to the nucleus and the presence of a free flagellum associated with the undulating membrane [2, 3]. The promastigote form is found in arthropod vectors, represented by insects of the phlebotomine group [4], while amastigote forms are found inside cells of the mononuclear phagocytic system of vertebrate hosts such as rodents, marsupials, bats, canids and humans [5, 6].

The severity of the disease is explained by the high virulence of its etiological agents, which results from the action of a set of functional molecules with intense biological activity in their hosts. The role of protease inhibitors in the development of pathogenic mechanisms was investigated [7]. These enzymes can be divided into endopeptidases and exopeptidases, depending on the site of hydrolysis. If the residue present at the catalytic site is taken into account, they are divided into metalloproteases, serine proteases, aspartic proteases and cysteine proteases [8]. In the genus *Leishmania*, cysteine proteases are the most abundant class of enzymes and are concentrated inside megasomes, where they act towards regulation of metabolic routes, cell differentiation in vertebrates and vectors, cell invasion and transposition of tissue barriers, degradation of hemoglobin and other hematopoietic proteins, evasion of the immune response, activation of inflammatory responses and programmed cell death [9].

Cathepsin L-*like* cysteine proteases in *Leishmania* have three isoforms, named cysteine protease A (CPA), cysteine protease B (CPB) and cysteine protease C (CPC). These are biochemically organized into four domains (predomain, prodomain, catalytic domain, C-terminal extension) that result from expression of a multigenic family arranged in tandem. In the specific case of *L. infantum,* two isoforms are expressed, the CPB isoform expressed in the promastigote forms found in the insect vectors and the CPA isoform, in which transcriptomic studies revealed a unique expression profile of the amastigote forms [10].

They are, therefore, used to construct phylogenetic inferences of close sequences and to resolve problems regarding polytomy and inferences of low support [11, 12]. Cathepsin genes have already been used for understanding phylogenetic relationships and as a target for making molecular diagnoses regarding other trypanosomatid species [13–15]. However, there are no studies that characterize this gene in *L. infantum* or that investigate it as a possible diagnostic marker that might help to solve recurrent problems regarding diagnostic investigation of this parasite within the clinical routine and in epidemiological investigations.

Despite the high importance of this disease, there is some difficulty in standardizing diagnostic methodologies with high predictive values for reservoir surveys. Making a direct diagnosis is invasive and laborious, and only low levels of sensitivity are reached [16–18]. The serological tests also have a series of technical limitations, such as low specificity values resulting from cross-reactions with other trypanosomatids, low concordance indices between different serological tests and lack of consensus regarding the nature and use of the antigenic product to be employed [19–22].

Thus, the objective of this study was to evaluate the CPA isoform of cathepsin L-*like* sequences as a marker for genetic analysis on intraspecific variability of *L. infantum* and as a marker for making molecular diagnoses on visceral leishmaniasis.

## Methods

### Leishmania isolates, DNA preparation, amplification and sequencing of cathepsin L-*like* gene

DNA from 44 *Leishmania* isolates (Table 1) was extracted from culture supernatants using the phenol-chloroform method and from primary samples (human blood, urine, conjunctival swabs from dogs and sandfly material) in accordance with the protocol established for the Purelink kit (Thermo Fisher Scientific Inc., 2012, USA).

**Table 1:** *Leishmania infantum* isolates, host, geographical origin and sequences of Cathepsin L-*like* employed in the phylogenetic analysis performed in this study

CBT^a^	Host	Geographical origin		Accession number^b^
01	*Canis familiaris*		SP	MH427793
12	*Canis familiaris*	Recreio	RJ	MH427794
13	*Canis familiaris*	Mangaratiba	RJ	MH427795
14	*Canis familiaris*	Ilha Grande	RJ	MH427796
15	*Canis familiaris*	Barra da Tijuca	RJ	MH427797
16	*Canis familiaris*	Recreio	RJ	MH427798
17	*Canis familiaris*	Cuiabá	MT	MH427799
18	*Canis familiaris*	Ilha de Guaratiba	RJ	MH427800
20	*Canis familiaris*	Cuiabá	MT	MH427801
22	*Canis familiaris*	Caucaia	CE	MH427802
23	*Canis familiaris*	Fortaleza	CE	MH427803
24	*Canis familiaris*	Jequié	BA	MH427804
25	*Canis familiaris*	Campo Grande	MS	MH427805
26	*Canis familiaris*		DF	MH427806
27	*Cerdocyon thous*		PA	MH427807
28	*Canis familiaris*	Teresina	PI	MH427808
29	*Canis familiaris*	Teresina	PI	MH427809
30	*Canis familiaris*	Uruguaiana	RS	MH427810
31	*Canis familiaris*	Uruguaiana	RS	MH427811
34	*Canis familiaris*	Petrolina	PE	MH427812
37	*Canis familiaris*	Petrolina	PE	MH427813
39	*Canis familiaris*	Santarém	PA	MH427814
40	*Canis familiaris*	Santarém	PA	MH427815
43	*Canis familiaris*	Santarém	PA	MH427816
44	*Canis familiaris*	Santarém	PA	MH427817
49	*Canis familiaris*	Campo Grande	MS	MH427818
50	*Canis familiaris*	Campo Grande	MS	MH427819
54	*Canis familiaris*	Teresina	PI	MH427820
55	*Canis familiaris*	Teresina	PI	MH427821
56	*Canis familiaris*	Campo Grande	MS	MH427822
57	*Canis familiaris*	Campo Grande	MS	MH427823
62	*Canis familiaris*	Petrolina	PE	MH427824
96	*Canis familiaris*	Caxias	MA	MH427825
105	*Canis familiaris*	Natal	RN	MH427826
106	*Canis familiaris*	Natal	RN	MH427827
107	*Canis familiaris*	Natal	RN	MH427828
108	*Canis familiaris*	Natal	RN	MH427829
124	*Canis familiaris*	Natal	RN	MH427830
125	*Canis familiaris*	Natal	RN	MH427831
126	*Canis familiaris*	Natal	RN	MH427832
153	*Canis familiaris*	São Domingos	MA	MH427833
179	*Canis familiaris*	Patos	PB	MH427834
211	*Homo sapiens*	Marília	SP	MH427835
212	*Homo sapiens*	Marília	SP	MH427836

Brazilian states: BA, Bahia; CE, Ceará; DF, Distrito Federal; MA, Maranhão; MS, Mato Grosso do Sul; MT, Mato Grosso; PA, Pará; PB, Paraíba; PE, Pernambuco; PI, Piauí; RJ, Rio de Janeiro; RN, Rio Grande do Norte; RS, Rio Grande do Sul; SP, São Paulo

^a^Coleção Brasileira de Tripanossomatídeos

^b^GenBank acession number

First of all, the extracted DNA samples were quantified and submitted to conventional polymerase chain reactions (PCR) using the specific primers to Mammalian Cytochrome B as a constitutive gene, to ensure the quality of the samples [23]. After this quality control, the DNA samples were submitted to the PCR reactions using high-fidelity Taq DNA polymerase with the specific primers designated for Cathepsin L-*like* CPA from *Leishmania* [24] which comprised a fragment of around 893 base pairs (bp).

All the isolates were included in the Brazilian Trypanosomatid Collection (Coleção Brasileira de Tripanossomatídeos, CBT) of the School of Veterinary Medicine of the University of São Paulo, Brazil.

### Phylogenetic analysis

The sequences obtained were aligned with sequences retrieved from GenBank using ClustalX [25] and were adjusted manually using GeneDoc [26] and then deposited in GenBank (Table 1). The cathepsin L-*like* CPA sequences were used to construct a phylogenetic tree using maximum parsimony, as implemented in PAUP version 4.0b10 [27] with 500 bootstrap replicates. Bayesian analysis was performed using MrBayes v3.1.2 [28] with 1,000,000 replicates. The first 25% of the trees represented burn-in, and the remaining trees were used to calculate Bayesian posterior probability.

### Standardization of *L. infantum*-specific assay based on cathepsin L-*like* protease

The aligned cathepsin L-*like* CPA gene sequences were used to search for consensus regions and to design specific primers for diagnosing *L. infantum*. The criteria used to define the primer pair were the guanine-to-cytosine ratio, formation of guanidine/cytosine (GC) staples, formation of self-homologies, self-dimer formation measured using the ΔG value, melting temperature and in silico specificity of the primers through BLASTn.

**Fig. 1:**
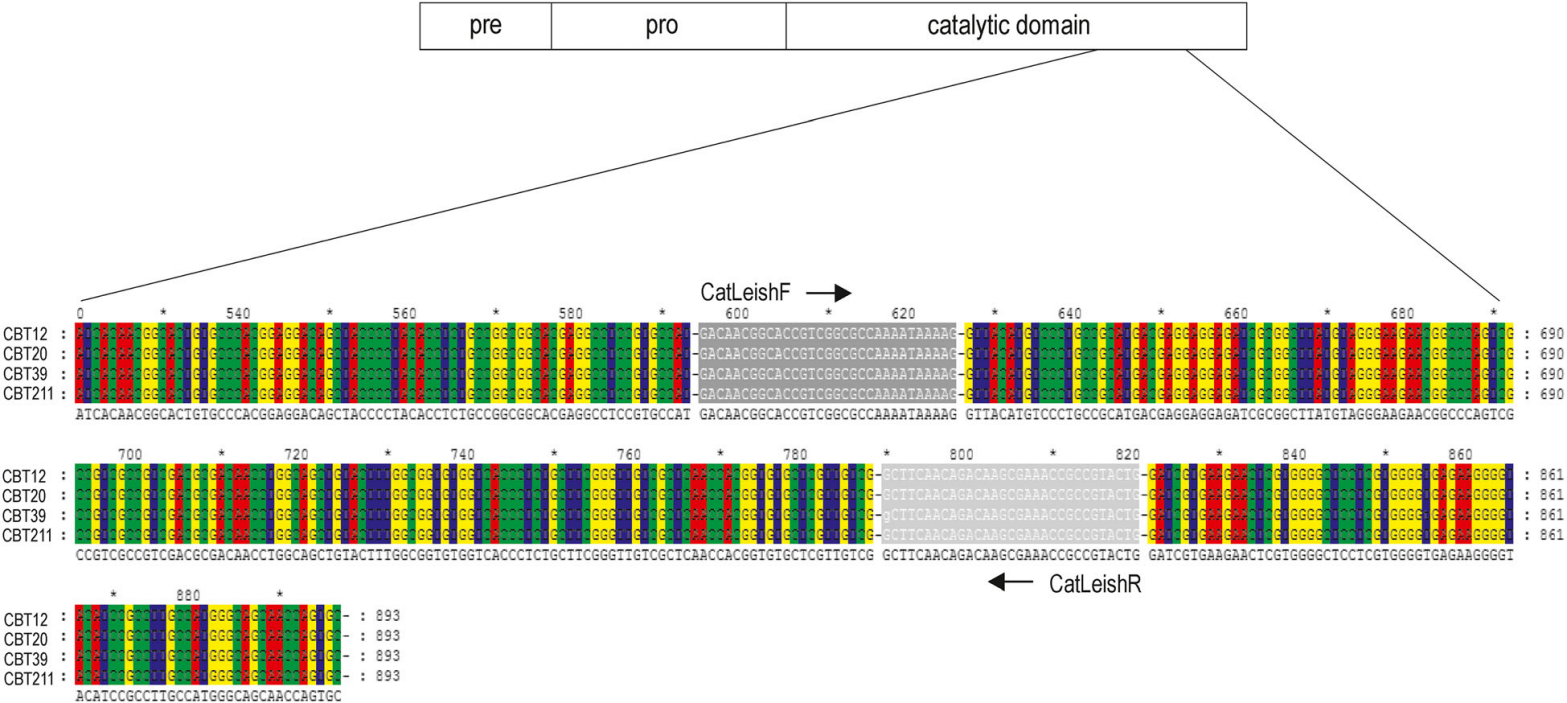
Alignment of cathepsin L-*like* CPA sequences from *Leishmania infantum*. Schematic representation of primers used for PCR amplification

A specific PCR procedure (designated CatLeish-PCR) was developed for amplification of 223 bp of genomic DNA from *L. infantum*, using the CatLeishF primer (5′ GACAACGGCACCGTCGGCGCCAAAATAAAAG 3′) and CatLeishR primer (5′ CAGTACGGCGGTTTCGCTTGTCTGTTGAAGC 3′) (Fig. 1). The standard conditions for amplification of the cathepsin L-*like* CPA sequences comprised 34 cycles of denaturation at 94 °C for 1 min, annealing at 64 °C for 1 min and extension at 72 °C for 45 s.

### Sensitivity and specificity assay on CatLeish-PCR

For sensitivity tests, DNA from *L. infantum* was serially diluted at the concentrations of 1 × 10^− 7^, 1 × 10^− 8^, 1 × 10^− 9^, 1 × 10^− 10^, 1 × 10^− 11^, 1 × 10^− 12^, 1 × 10^− 13^, 1 × 10^− 14^ and 1 × 10^− 15^ ng/μl.

Specificity tests were performed on DNA samples from other parasite species in the genus *Leishmania*, including: *Leishmania (Viannia) braziliensis*, *Leishmania (Viannia) guyanensis*, *Leishmania (Viannia) naiffi*, *Leishmania (Leishmania) amazonensis*, *Leishmania (Leishmania) mexicana*, *Leishmania (Sauroleishmania) gymnodactyli*, *Leishmania (Sauroleishmania) adleri*, *Leishmania (Sauroleishmania) tarentolae* and *Leishmania (Mundinia) enriettii.* In addition, the following species in the genus *Trypanosoma* were tested: *Trypanosoma dionisii*, *Trypanosoma terrestris*, *Trypanosoma cruzi*, *Trypanosoma cruzi marinkellei*, *Trypanosoma theileri* and *Trypanosoma gennarii*; along with two other pathogenic species that are common in dogs: *Babesia canis* and *Ehrlichia canis*.

### Application of CatLeish-PCR to clinical samples

CatLeish-PCR was tested on a panel of clinical samples of purified DNA obtained from dogs in endemic and non-endemic regions in São Paulo state, Brazil. In addition, blood samples (prepared on filter paper), conjunctival swabs and urine samples from dogs that were known to be positive in the parasitological test were used. DNA samples obtained from human patients blood from Marília, São Paulo state and DNA extracted from sandflies caught in Bom Jesus dos Perdões, São Paulo state were also tested in pools. All samples were further tested using the ITS rDNA marker [29].

## Results

**Fig. 2:**
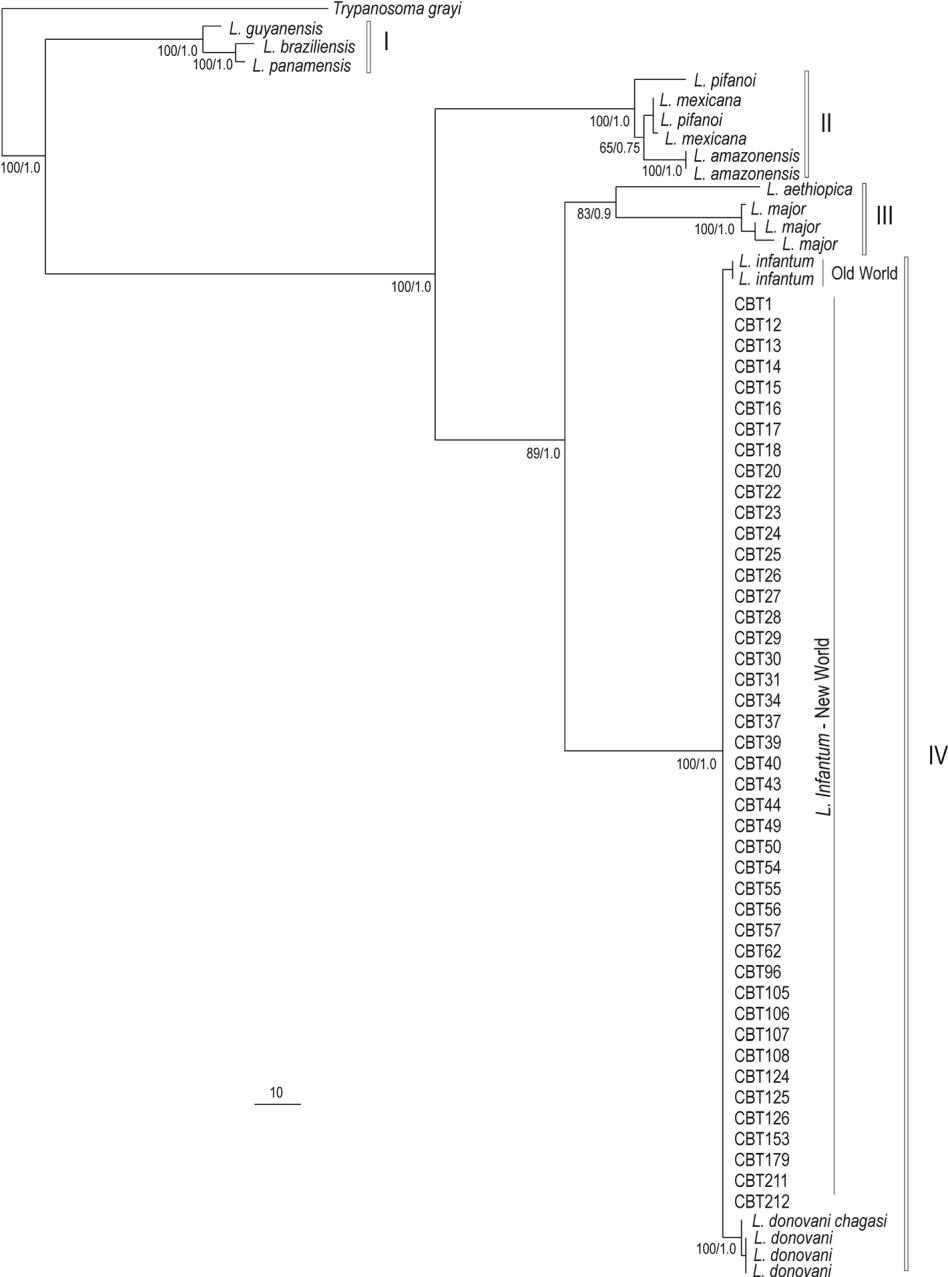
Dendrogram based on 44 cathepsin L-*like* sequences from *Leishmania infantum*, which was used for making maximum parsimony and Bayesian inferences with 892 characteristics. Numbers at nodes are the support values for the major branches (bootstrap/posterior probability; 500 replicates). The sequences obtained in the present study are underlined

Forty-four sequences were obtained from cathepsin L-*like* CPA and they were all identical, without polymorphism, and presented 99% similarity with cysteine peptidase isoform A of *Leishmania infantum* (XM_001465076.1) from Europe. The sequences of cathepsin L-like CPA from *L. infantum* were identical and all isolates were clustered together (100% bootstrap/100% posterior probability and 100% similarity) (Fig. 2).

*Leishmania* species were aggregated in a monophyletic group (Fig. 2). The different species of the genus *Leishmania* were segregated into four groups: I. *Leishmania* species with mucocutaneous clinical manifestations, including *L. guyanensis*, *L. braziliensis* and *L. panamensis* (0.1% divergence of sequences and 100% bootstrap and 1.0 posterior probability); II. Species with cutaneous manifestations, including *L. pifanoi*, *L. mexicana* and *L. amazonensis* (0.52% divergence of sequences and 100% bootstrap and 1.0 posterior probability); III. *Leishmania* species causing cutaneous “oriental sore”, including *L. major* and *L. aethiopica* (1.53% divergence of sequences and 100% bootstrap and 1.0 posterior probability); and IV. New and Old-World species of visceral leishmaniasis (0.14% divergence of sequences and 100% bootstrap and 1.0 posterior probability), separated according to the geographical origin of the isolates (Fig. 2).

The in silico analyses on the primers proposed for the specific PCR did not indicate any possibility of formation of GC staples, self-dimers and auto-homologies. The CatLeishF primer showed a GC ratio of 54.5% with an estimated melting temperature of 66.2 °C, whereas the CatLeishR primer had a GC ratio of 57.6% with a melting temperature corresponding to 65.5 °C. In BLASTn analysis, these primers were specific for *L. infantum*, with no homology containing sequences from other species deposited in the database.

**Fig. 3:**
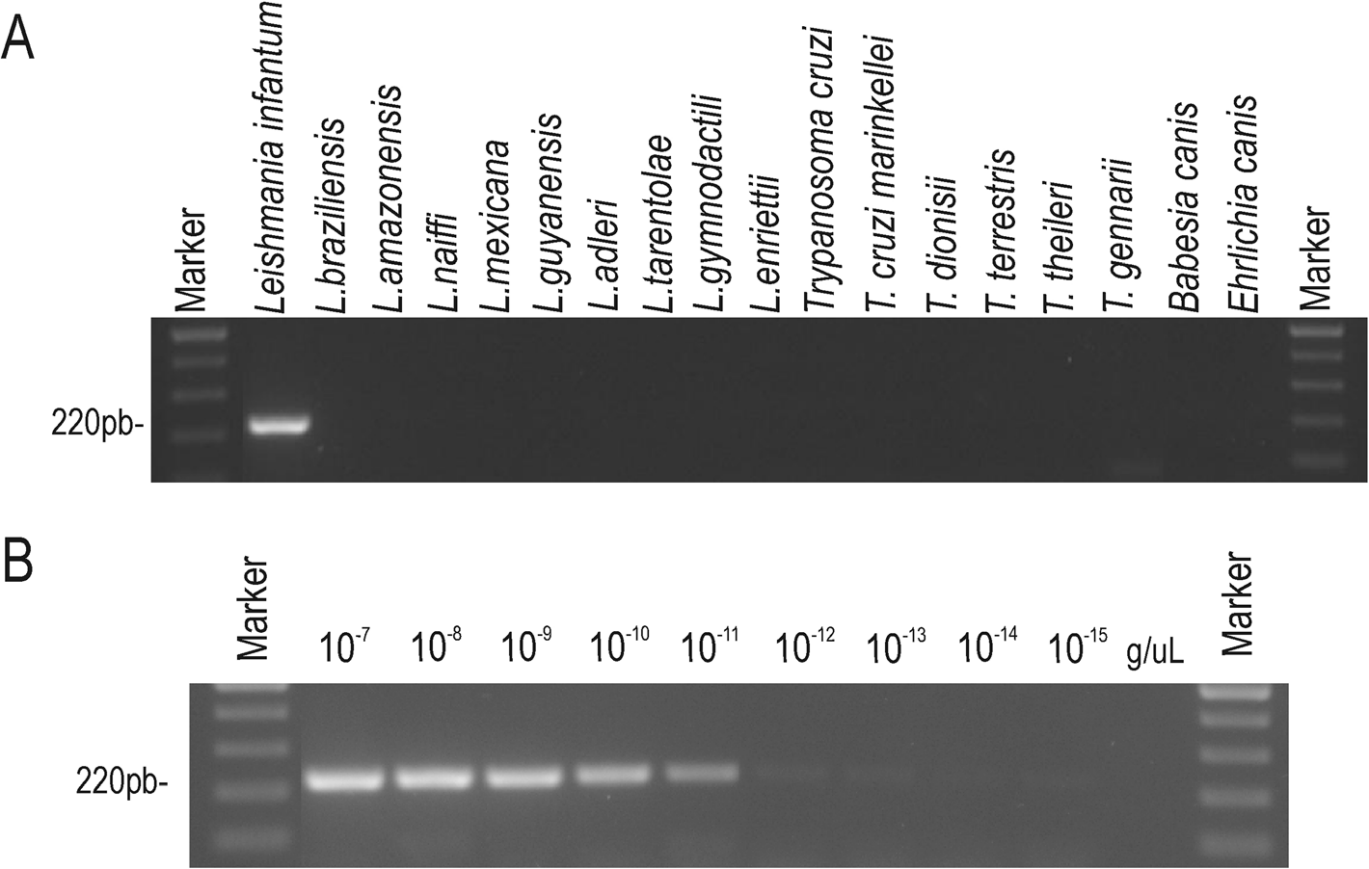
CatLeish-PCR for diagnosis of *Leishmania infantum* based on Cathepsin L-like sequences. **a** Specificity analysis using DNA, *Leishmania* and *Trypanosoma* sequences and other hemoparasites from dogs (*Babesia* and *Ehrlichia*). **b** Sensitivity analysis using DNA from *Leishmania infantum* in different concentrations

CatLeish-PCR reactions with genomic DNA from *L. infantum* isolates demonstrated that there was a correct amplification, since amplicon sequencing revealed in BLASTn a percentage of 99% similarity to deposited sequences of cysteine protease genes from European strains of *L. infantum* (XM_001465076.1) and identical to American *L. infantum* sequences obtained in this study. Sensitivity measurement demonstrated that the markers were efficient at amplifying the target DNA and forming detectable bands at concentrations of up to 10^− 11^ ng/μl (Fig. 3).

Different species of the genus *Leishmania* were tested and there was no cross-amplification with any of the species epidemiologically associated with the cutaneous or tegumentary leishmaniasis complex in Brazil. When the molecular diagnosis was tested against hemoparasites belonging to the genera *Babesia*, *Ehrlichia* and *Trypanosoma*, there was no amplification (Fig. 3).

The use of CatLeish-PCR in the panel of clinical samples revealed that frequency of positive dogs was 49.03%, thus contrasting with the 14.42% achieved through using primers for the ribosomal ITS gene. All samples that were positive in the reactions for the ITS gene were positive in reactions for the cathepsin gene. In the human samples, 6.25% were positive, whereas in samples from sandflies, the positivity was 14.28% in pools (Table 2).

**Table 2:** Positivity of naturally infected biological samples based on Cathepsin L-*like *of *Leishmania infantum* (CatLeish-PCR) marker

Samples (number)	Positives samples (%)	
	CatLeish-PCR	ITS
Dogs blood (208)	49,03 (102)	14,42 (30)
Dogs urine (3)	33 (1)	
Dogs conjuntival swab (4)	50 (2)	
Dogs blood in filter paper (4)	100 (4)	
Human blood (50)	6,25 (3)	
Sand flies (21)	14,28 (3)	

The samples from parasitologically positive dogs showed different rates of positivity according to the material tested. Urine samples showed 33% positivity, conjunctival swabs 50%, samples preserved on filter paper 100% and whole blood samples 100%.

The CatLeish-PCR was replicated several times during standardization with DNA samples from American *L. infantum* isolates, and subsequently technical replication was tested with clinical samples. Regardless of the samples tested, the technical replication and amplification standard were maintained (Additional file 1).

## Discussion

The phylogenetic inferences from maximum parsimony and Bayesian analyses revealed a pattern of high similarity for *L. infantum* isolates, as observed in inferences based on SSUrDNA and gGAPDH genes [30]. The segregation of *L. infantum* from Europe and America corroborates the notion that this species was introduced to the Americas during the colonization period [30].

This absence of divergence, even among isolates from biomes with different climates, rainfall, vegetation cover and availability of hosts and arthropod vectors, corroborates the theory that this parasite is not autochthonous to the Americas [31]. This finding indicates that the event leading to introduction of this parasite to the New World was recent. It can therefore probably be dated to the process of Ibero-American colonization that began in the fifteenth century, caused by the arrival of infected dogs and rodents [32]. Focusing only on a few specific genes we can speculate that this short evolutionary time interval would be insufficient for the appearance and fixation of possible mutations in the population.

These results refute the hypothesis of a scenario in which the parasite responsible for cases of visceral leishmaniasis is native to the Americas [30–33]. Thus, the argument that the parasite is autochthonous in that it is adapted to parasitism in other wild fauna reservoirs can be countered through attributing this adaptation to the eclectic dietary habits of the vector insects [34].

The phylogenetic analysis on the cathepsin L-*Like* CPA gene showed clustering that reflects the distinct clinical manifestations of *Leishmania* infection, and it was similar to amino acid analyses on cysteine proteases [24]. In addition, the topology generated using the cathepsin L-*like* gene also corroborates data generated using ribosomal spacers and cytochrome B [35, 36].

The inferences correctly solved the differentiation of the species that are considered to be the etiological agent of tegumentary forms of the disease. One of the clades grouped *L. guyanensis*, *L. panamensis* and *L. braziliensis*, and this reflected the findings of studies in which, analyzing otorhinolaryngological conditions, most of the etiology of mucocutaneous leishmaniasis was attributed to *L. braziliensis* and *L. panamensis*, as well as results in which clinical situations of mucosal involvement were attributed to *L. guyanensis* [37, 38].

A second group included *L. amazonensis* and *L. mexicana*, species that have been widely associated with cutaneous manifestations of tegumentary leishmaniasis in the New World [39–43]. The *L. pifanoi* sequences were positioned in this same group. Although this species has lower epidemiological impact regarding disease transmission, it belongs to the *L. mexicana* complex [44]. Therefore, this result corroborates the data demonstrating similarity among the species of this complex, with regard to proposing specific markers for kinetoplast DNA (kDNA) [45].

These results confirm that the cathepsin L-*like* CPA gene is a good marker for phylogenetic positioning of species of the genus *Leishmania*, which suggests that this gene may be a good target for making the molecular diagnosis of leishmaniasis. This would enable satisfactory differentiation between the clinical forms of the disease, including among the variants of the tegumentary forms, which may aid in prescribing therapy, establishing the medical prognosis for the disease and mitigating the recurrent problems of specific diagnosis. We agree that the number of isolates collected in this work is sufficient to portray the dispersal of the parasite in Brazil.

Cathepsin gene-based assays have shown excellent results with regard to diagnosing the following *Trypanosoma* species: *T. vivax* [13], *T. congolense* [46], *T. theileri* [47], *T. cruzi* [48] and *T. rangeli* [49]. However, the low numbers of copies of this gene in the genome are very specific and especially sensitive [13].

Cathepsin L-*like* protease is differentially expressed in different forms of the parasite [50, 51]. The CPA isoform is preferentially expressed in amastigote forms. In vertebrates (both human and animal hosts), amastigotes are the replicative form and cathepsin L-*like* CPA is continuously expressed [24]. The expression pattern of cathepsin L-*like* CPA and the presence of immunogenic epitopes may indicate it as an interesting target in diagnostic-serological tests, which are preferentially used in the clinical routine [25].

The diagnostic marker based on the cathepsin L-*like* CPA gene has shown high sensitivity and was specific for *L. infantum*, which enables direct use in clinical samples both in non-endemic areas with imported cases and in endemic areas in which different species of *Leishmania* may be circulating. Thus, it has been proven to be effective for epidemiological surveys on human hosts, animal reservoirs and arthropod vectors. Another advantage that makes CatLeish-PCR feasible is that there is no need for complementary restriction enzyme digestion steps or use of robust high-resolution melting equipment for identifying *L. infantum*, which makes the method less costly and less cumbersome than other methodologies that are available [52].

In comparison with the markers for the ITS gene, CatLeish-PCR demonstrated higher prevalence of leishmaniasis, which thus corroborated the diagnostic sensitivity of the marker. The marker was effective in making the diagnosis, both from positive swab samples from conjunctival lesions and from blood samples fixed on filter paper, reinforcing the high sensitivity of CatLeish-PCR. The positive animals in the parasitological examination were also positive when submitted to molecular diagnosis based on the cathepsin L-*like* gene, except for the urine samples given the low amount of DNA in two animals. This versatility is important because samples fixed on filter paper are easier to transport and store, given their stability, as well as exempt invasive collection methodologies [29].

The diagnostic method works very well for blood samples and even though the number of samples from other sources is reduced, the method borrows our hypothesis of good molecular marker for diagnosis in biological samples with DNA. Amplification was observed even in samples extracted from urine, whose DNA concentration is considerably reduced, corresponding only to fragments of the dead parasite excreted in the urinary system [53]. It should be noted that even with low levels of positivity in the assays using biological samples as a template, we still support our indication of the use of the Cathepsin gene for PCR diagnosis, since even in biological samples where the genetic material of the parasites is scarce or damaged, we obtained satisfactory amplifications. Considering this, CatLeish-PCR may have potential for monitoring the remission of infection in patients with visceral leishmaniasis and as an indicator of the efficacy of possible therapeutic approaches [54].

The results presented here allow us to propose that CatLeish-PCR is a tool that can be used for diagnosing visceral leishmaniasis. It surmounts the recurrent problems of low sensitivity that direct visualization methodologies present and those of low specificity and agreement among the serological methods that have preferentially ben recommended for diagnosing this disease [55]. The molecular diagnostic assays used and described in the literature have low sensitivity and specificity. Markers based on the cytochrome b gene are non-specific [56]. Markers based on genes present in the kinetoplast have high sensitivity, but their specificity is low [57, 58]. Markers based on ribosomal genes are specific, but not very sensitive [59]. Other assays such as restriction fragment length polymorphism (PCR-RFLP) have been standardized, but these raise the cost of making the diagnosis or still require sequencing of the product [60–62].

Epidemiological studies are important for recognizing active transmission cycles or introduction of parasites into new areas. Therefore, precise diagnostic methods are essential for diagnosing the presence of a parasite that is still emerging in many regions. Moreover, making a precise diagnosis minimizes the need to destroy dogs, which are the main reservoirs, given that in some countries this prophylactic measure used in combating human leishmaniasis.

## Conclusion

This is the first work that characterizes the CPA isoform of the L-*like* cathepsin gene of *L. infantum*, demonstrating the lack of genetic variability among the Brazilian isolates of *L. infantum*. We propose that the gene studied here be an efficient phylogenetic marker for parasites of the genus *Leishmania*, and also the developed CatLeish-PCR has been shown to be sensitive and specific for the effective clinical diagnosis of this zoonosis in dogs and humans.

## Supplementary information

**Additional file 1.** Amplification patterns of CatLeish-PCR for diagnosis of *Leishmania infantum* in samples from dogs, humans and sand flies.

## Data Availability

The accession numbers of GenBank from sequences obtained in this study are described in Table 1.

## Abbreviations

C: Cytosine
CBT: Coleção Brasileira de Tripanossomatídeos
CPA: Cysteine protease A
CPB: Cysteine protease B
CPC: Cysteine protease C
DNA: Deoxyribonucleic acid
G: Guanidine
ITS: Internal transcribed spacer
kDNA: kinetoplast deoxyribonucleic acid
PCR: Polymerase chain reaction
PCR-RFLP: Restriction fragment length polymorphism
