# Supplementary figures and images for: Exploring Leishmania infantum cathepsin as a new molecular marker for phylogenetic relationships and visceral leishmaniasis diagnosis
Source: BMC Infect Dis. 2019 Oct 28;19:895. doi: 10.1186/s12879-019-4463-8 (PMC6819481; doi:10.1186/s12879-019-4463-8)

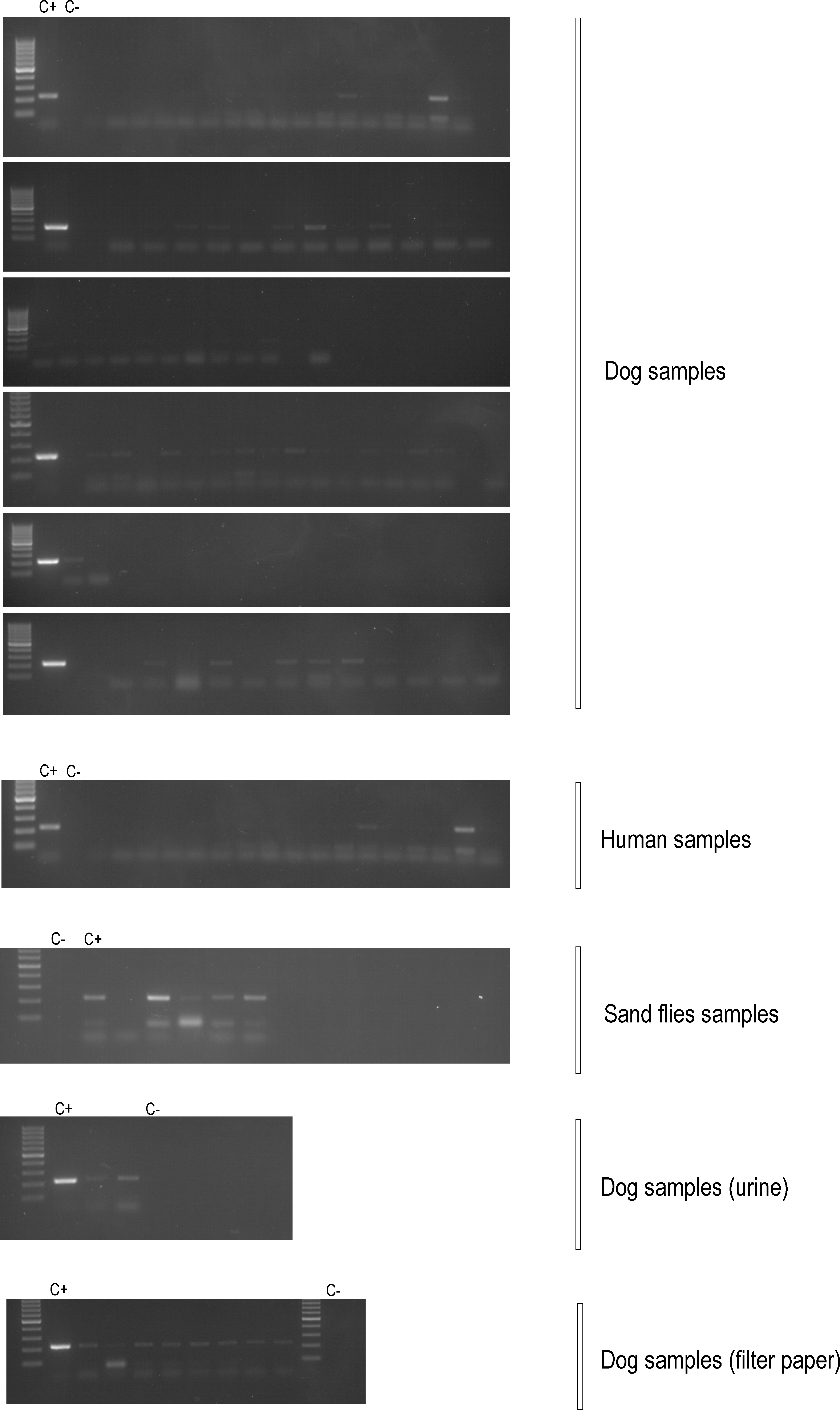

Supplement: Supplementary file 1 — Additional file 1. Amplification patterns of CatLeish-PCR for diagnosis of Leishmania infantum in samples from dogs, humans and sand flies. [file 12879_2019_4463_MOESM1_ESM.jpg]
